# Association of Active Postnatal Care With Infant Survival Among Periviable Infants in the US

**DOI:** 10.1001/jamanetworkopen.2022.50593

**Published:** 2023-01-19

**Authors:** Emani R. Silva, Vivek V. Shukla, Rachel Tindal, Waldemar A. Carlo, Colm P. Travers

**Affiliations:** 1 University of Alabama at Birmingham School of Medicine, Birmingham; 2 Department of Pediatrics, University of Alabama at Birmingham, Birmingham

## Abstract

**Importance:**

Active postnatal care has been associated with center differences in survival among periviable infants. Regional differences in outcomes among periviable infants in the US may be associated with differences in active postnatal care.

**Objective:**

To determine if regions with higher rates of active postnatal care will have higher gestational age-specific survival rates among periviable infants.

**Design, Setting, and Participants:**

This cohort study included live births from 22 to 25 weeks’ gestation weighing 400 to 999 g in the US Centers for Disease Control and Prevention (CDC) WONDER 2017 to 2020 (expanded) database. Infants with congenital anomalies were excluded. Active postnatal care was defined using the CDC definition of abnormal conditions of newborn as presence of any of the following: neonatal intensive care unit (NICU) admission, surfactant, assisted ventilation, antibiotics, and seizures. Data were analyzed from August to November 2022.

**Main Outcomes and Measures:**

Regional gestational age-specific survival rates were compared with rates of active postnatal care in the 10 US Health and Human Services regions using Kendall τ test.

**Results:**

We included 41 707 periviable infants, of whom 32 674 (78%) were singletons and 19 467 (46.7%) were female. Among those studied 34 983 (83.9%) had evidence of active care, and 26 009 (62.6%) survived. Regional rates of active postnatal care were positively correlated with regional survival rates at 22 weeks’ gestation (*r*_τ_[8] = 0.56; *r*^2^ = 0.31; *P* = .03) but the correlation was not significant at 23 weeks’ gestation (*r*_τ_[8] = 0.47; *r*^2^ = 0.22; *P* = .07). There was no correlation between active care and survival at 24 or 25 weeks’ gestation. Regional rates of both NICU admission and assisted ventilation following delivery were positively correlated with regional rates of survival at 22 weeks’ gestation (both *P* < .05). Regional rates of antenatal corticosteroids exposure were also positively correlated with regional rates of survival at 22 weeks’ gestation (*r*_τ_[8] = 0.60; *r*^2^ = 0.36; *P* = .02).

**Conclusions and Relevance:**

In this cohort study of 41 707 periviable infants, regional differences in rates of active postnatal care, neonatal intensive care unit admission, provision of assisted ventilation and antenatal corticosteroid exposure were moderately correlated with survival at 22 weeks’ gestation. Further studies focused on individual-level factors associated with active periviable care are warranted.

## Key Points

**Question:**

Do regions with higher rates of active postnatal care have higher gestational age-specific survival rates among periviable infants in the US?

**Findings:**

In this cohort study of 41 707 periviable infants, regional differences in active postnatal care, including assisted ventilation and neonatal intensive care unit admission, were moderately correlated with differences in survival at 22 weeks’ gestation.

**Meaning:**

These findings suggest that differences in the provision of active treatment were associated with interregional variation in survival at 22 weeks’ gestation.

## Teaser

This cohort study evaluates the association of active postnatal care with infant survival among periviable infants in the US.

## Introduction

The decision of whether to initiate active care in periviable infants born at 22 to 25 weeks’ gestation is influenced by several factors, including the fear of adverse outcomes, individual bias, local and national policies, medical ethics, and communication between parents and caregivers.^1,2,3,4,5^ In the US, professional societies recommend an individualized approach^3^ and consideration of active perinatal care, including antenatal corticosteroids^6,7^ and active postnatal care^8^ if resuscitation is desired.^9^ The lower threshold of viability is currently considered to be 22 weeks’ gestation.^8^ Differences in practices regarding the initiation of active postnatal treatment among periviable infants explained much of the variation in survival rates among centers included in the Eunice Kennedy Shriver National Institute of Child Health and Human Development Neonatal Research Network.^8^ Although it is known that there is geographic variation in extremely preterm infant mortality rates within the US,^10^ the factors driving these differences at a population level are not clear.

Data from Sweden suggest that differences in regional infant mortality rates among periviable infants were associated with differences in survival in the first 12 hours after birth.^11^ There were also differences in regional perinatal care practices, including antenatal corticosteroids, cesarean delivery, surfactant, intubation at birth, and admission to a neonatal intensive care unit. Higher rates of active perinatal care practices were associated with lower infant mortality rates among periviable infants.^12^ Furthermore, rates of active perinatal and postnatal care among periviable infants may be higher in the Southern and Midwestern regions compared with the Northeastern and Western regions of the US.^13^ Therefore, it is possible that regional differences in outcomes among periviable infants in the US are associated with differences in active care. We hypothesized that regions with higher rates of active postnatal care among infants at the lowest gestational age categories will have lower gestational age-specific infant mortality rates. We further hypothesized that regions with higher rates of antenatal corticosteroids and cesarean delivery will have higher rates of survival among infants in the lowest gestational age categories.

## Methods

This cohort study used collapsed regional-level data from the US Centers for Disease Control and Prevention (CDC) Wide-ranging Online Data for Epidemiologic Research (WONDER) linked live birth and infant death expanded database from 2017 to 2020.^14^ Live birth and infant death data from all 57 vital statistic jurisdictions of the Vital Statistics Cooperative Program were included. Gestational age was calculated using an obstetrician’s estimate when available and last menstrual period if not available. The following gestational age subgroups were included: 22 weeks’, 23 weeks’, 24 weeks’, and 25 weeks’ gestation. We included infants with a birth weight from 400 to 999 g. The 400 g lower limit is less than the 10th percentile for infants at 22 weeks’ and 0 days’ gestation while the 999 g upper limit is approximately the 90th percentile for weight at 25 weeks’ and 6 days’ gestation.^15^ Stillbirths and infants who were not linked to a gestational age or birth weight were excluded. Infants with congenital anomalies and those born outside of a hospital/birth center were also excluded from this study. Individual patient data are not available in the data set and birth or death counts with less than 10 infants per region were not available per National Center for Health Statistics subnational geography data use restrictions. The study was reviewed by the institutional review board at the University of Alabama and designated not human participants research as the data are publicly available and deidentified; therefore, informed consent was not required. We followed the Strengthening the Reporting of Observational Studies in Epidemiology (STROBE) reporting guideline for cohort studies.^16^

### Definitions

The 10 US regions in this study were defined according to the Department of Health and Human Services (HHS) as follows: (1) Connecticut, Maine, Massachusetts, New Hampshire, Rhode Island, and Vermont; (2) New Jersey, New York, Puerto Rico, and US Virgin Islands; (3) Delaware, District of Columbia, Maryland, Pennsylvania, Virginia, and West Virginia; (4) Alabama, Florida, Georgia, Kentucky, Mississippi, North Carolina, South Carolina, and Tennessee; (5) Illinois, Indiana, Michigan, Minnesota, Ohio, and Wisconsin; (6) Arkansas, Louisiana, New Mexico, Oklahoma, and Texas; (7) Iowa, Kansas, Missouri, and Nebraska; [8] Colorado, Montana, North Dakota, South Dakota, Utah, and Wyoming; (9) America Samoa, Arizona, California, Commonwealth of the Northern Mariana Islands, Federated States of Micronesia, Guam, Hawaii, Nevada, Republic of Palau, and Republic of the Marshall Islands; and (10) Alaska, Idaho, Oregon, and Washington.

Active postnatal care was defined as using 1 or more of the following according to the CDC definition of “abnormal conditions of newborn”: neonatal intensive care unit (NICU) admission, surfactant replacement therapy, assisted ventilation (either immediately following delivery and/or if required for more than 6 hours), antibiotics for suspected neonatal sepsis, and seizures. This definition was chosen on the basis that with the exception of seizures, which is a fairly infrequent diagnosis among periviable infants but could not be removed from the definition due to database limitations, the other conditions represent process measures suggesting that active postnatal care had been provided. For example, at 22 weeks’ gestation an infant receiving active postnatal care would routinely be intubated for surfactant and mechanical ventilation in the delivery room followed by admission to the neonatal intensive care unit where antibiotics would be started for suspected sepsis. Infant mortality was defined as death within 365 days after birth. Race was defined per CDC maternal single race (American Indian or Alaska Native, Asian, Black or African American, Native Hawaiian or Pacific Islander, White, and more than 1 race) and Hispanic origin categories. Race was included as a baseline characteristic in this study as race is associated with differences in preterm birth and infant mortality rates. Insurance status was defined according to the payment method at the time of delivery. Early prenatal care was defined as prenatal care beginning in the first to third months of pregnancy. Higher-risk pregnancy was defined according to presence of any diabetes, hypertensive disorders, previous preterm birth, fertility treatments or assisted reproduction, or previous cesarean delivery.

### Statistical Analysis

The primary outcome measure evaluated gestational age-specific survival rates by rates of active postnatal care in the 10 HHS regions. Secondary outcomes measures were evaluated by prespecified gestational age categories. Variables included regional rates of gestational age-specific survival, regional rates of active postnatal care and the individual measures of active postnatal care, regional rates of key antenatal care practices including early prenatal care, antenatal corticosteroids, and cesarean section. We also examined whether regional gestational age-specific survival at lower gestational age categories was associated with gestational age-specific survival among infants in higher gestational age categories. Missing data were not imputed. We used Kendall τ test to determine the strength of the association between variables. We created heat maps showing the strength of correlations between pairs of available variables at each gestational age. We used 2-sided χ^2^ tests or Fischer exact tests to compare the differences in demographic and clinical characteristics, and significance was set at *P* < .05. R version 4.0.0 (R Project for Statistical Computing) and extension packages dplyr, ggplot2, and base R were used for all analyses. Data were analyzed from August to November 2022.

## Results

### Infant and Maternal Characteristics

We included 41 707 periviable infants, of whom 32 674 (78%) were singletons and 19 467 (46.7%) were female (Table 1). Among those included in this study, 5728 infants (13.7%) were delivered at 22 weeks’, 9629 infants (23.1%) at 23 weeks’, 12 714 infants (30.5%) at 24 weeks’, and 13 636 infants (32.7%) at 25 weeks’ of gestational age. Overall, 12 383 infants (29.7%) were non-Hispanic Black and 7876 infants (18.9%) were Hispanic. Medicaid public insurance covered 17 361 births (41.6%). Approximately 28 977 mothers (69.5%) had established prenatal care by 3 months and 6442 pregnancies (15.4%) were considered higher-risk.

**Table 1.** Infant/Maternal Baseline Clinical and Demographic Characteristics Present at Birth by HHS Region, 2017 to 2020 (N = 41 707)

Characteristic	No. (%)										
	All regions	HHS 1	HHS 2	HHS 3	HHS 4	HHS 5	HHS 6	HHS 7	HHS 8	HHS 9	HHS 10
Gestational age, wk											
22	5728 (13.7)	167 (14.5)	381 (11.6)	618 (14.8)	1410 (13.6)	953 (14.3)	924 (13.8)	246 (14.1)	155 (13.1)	718 (13.6)	156 (13.8)
23	9629 (23.1)	229 (19.8)	756 (22.9)	947 (22.7)	2483 (23.9)	1523 (22.9)	1568 (23.4)	420 (24.1)	284 (23.9)	1165 (22.1)	254 (22.5)
24	12 714 (30.5)	357 (30.9)	1046 (31.7)	1268 (30.4)	3135 (30.1)	2039 (30.7)	2004 (29.8)	517 (29.7)	343 (28.9)	1663 (31.6)	342 (30.3)
25	13 636 (32.7)	402 (34.8)	1115 (33.8)	1335 (32.0)	3374 (32.4)	2128 (32.0)	2219 (33.0)	560 (32.1)	404 (34.1)	1722 (32.7)	377 (33.4)
Female sex^a^	19 467 (46.7)	538 (46.6)	1531 (46.4)	1931 (46.3)	4878 (46.9)	3122 (47.0)	3201 (47.7)	822 (47.2)	533 (44.9)	2404 (45.6)	507 (44.9)
Singleton	32 674 (78.3)	891 (77.1)	2508 (76.0)	3250 (78.0)	8238 (79.2)	5130 (77.2)	5320 (79.2)	1341 (76.9)	918 (77.4)	4197 (79.7)	881 (78.0)
Medicaid	17 361 (41.6)	414 (35.8)	1231 (37.3)	1617 (38.8)	4790 (46.0)	2684 (40.4)	3033 (45.2)	668 (38.3)	345 (29.1)	2152 (40.9)	427 (37.8)
Higher-risk pregnancy	6442 (15.4)	155 (13.4)	479 (14.5)	625 (15.0)	1658 (15.9)	1100 (16.6)	1092 (16.3)	290 (16.6)	120 (10.1)	754 (14.3)	169 (15.0)
Early prenatal care	28 977 (69.5)	928 (80.3)	2339 (70.9)	2776 (66.6)	7047 (67.7)	4552 (68.5)	4362 (65.0)	1197 (68.7)	828 (69.8)	4185 (79.4)	763 (67.6)
Race/ethnicity											
Black	12 383 (29.7)	253 (21.9)	1083 (32.8)	1564 (37.5)	4501 (43.3)	1993 (30.0)	1805 (26.9)	389 (22.3)	88 (7.4)	617 (11.7)	90 (8.0)
Hispanic	7876 (18.9)	246 (21.3)	744 (22.6)	491 (11.8)	1152 (11.1)	627 (9.4)	1938 (28.9)	170 (9.8)	243 (20.5)	2063 (39.2)	202 (17.9)

Abbreviation: HHS, Department of Health and Human Services.

^a^ Data for male sex are not available.

### Active Postnatal Care

Evidence of active postnatal care was seen in 34 983 infants (83.9%). Rates of active care varied by region and by gestational age (Table 2). Active care intervention was more common in infants born at 25 weeks’ gestation compared with those born at 22 weeks’ gestation (12 744 infants [93.5%] vs 2378 infants [41.5%]). At 25 weeks’ gestation, rates of active postnatal care ranged from 1517 infants in region 9 (88.1%) to 394 infants in region 8 (97.5%). At 22 weeks’ gestation, rates of active postnatal care ranged from 32 infants in region 8 (20.6%) to 137 infants in region 7 (55.7%). The frequency of individual active care interventions varied by region and gestational age as well (Table 2). NICU admission was reported in 12 419 infants at 25 weeks’ gestation (91.1% [range 85.6% to 95.5%]) and 2145 infants at 22 weeks’ gestation (37.4% [range 17.4% to 48.8%]).

**Table 2.** Regional Rates of Measures of Survival, Active Postnatal Care, and Perinatal Care Among Periviable Infants Overall and by Gestational Age From 22 to 25 Weeks, 2017 to 2020 (N = 41 707)

Outcome by gestational age in wk	No. (%)										
	All regions	HHS 1	HHS 2	HHS 3	HHS 4	HHS 5	HHS 6	HHS 7	HHS 8	HHS 9	HHS 10
Survival overall	26 099 (62.6)	723 (62.6)	2055 (62.3)	2589 (62.1)	6612 (61.0)	4049 (65.4)	4389 (63.1)	1100 (54.0)	640 (61.6)	3247 (61.6)	695 (61.6)
22	1086 (18.9)	28 (16.8)	45 (11.8)	113 (18.3)	288 (20.4)	164 (17.3)	235 (25.4)	68 (27.6)	13 (8.4)	103 (14.3)	29 (18.6)
23	4887 (50.8)	103 (44.5)	359 (47.5)	474 (50.1)	1305 (52.6)	750 (49.2)	890 (56.7)	229 (54.5)	103 (36.3)	546 (46.9)	128 (50.4)
24	8986 (70.7)	248 (69.5)	741 (70.8)	896 (70.7)	2263 (72.2)	1416 (69.4)	1441 (71.9)	354 (68.5)	211 (61.5)	1173 (70.1)	243 (71.1)
25	11 140 (81.7)	344 (85.6)	910 (81.6)	1106 (82.8)	2756 (81.7)	1719 (80.8)	1823 (82.2)	449 (80.2)	313 (77.5)	1425 (82.8)	295 (78.2)
Active postnatal care overall	34 983 (83.9)	932 (80.7)	2841 (86.1)	3463 (83.1)	8979 (86.3)	5668 (85.3)	5700 (84.9)	1556 (89.3)	950 (80.1)	4011 (76.1)	883 (78.2)
22	2378 (41.5)	56 (33.5)	141 (37.0)	206 (33.3)	721 (51.1)	399 (41.9)	437 (47.3)	137 (55.7)	32 (20.6)	204 (28.4)	45 (28.8)
23	8128 (84.4)	185 (80.8)	654 (86.5)	796 (84.1)	2182 (87.9)	1324 (86.9)	1363 (86.9)	376 (89.5)	206 (72.5)	860 (73.8)	182 (71.7)
24	11 733 (92.3)	323 (90.5)	980 (93.7)	1181 (93.1)	2913 (92.9)	1917 (94.0)	1858 (92.7)	500 (96.7)	318 (92.7)	1430 (86.0)	313 (91.5)
25	12 744 (93.5)	368 (91.5)	1066 (95.6)	1280 (95.9)	3163 (93.7)	2028 (95.3)	2042 (92.0)	543 (97.0)	394 (97.5)	1517 (88.1)	343 (91.0)
NICU admission overall	33 666 (80.7)	858 (74.3)	2763 (83.8)	3297 (79.1)	8694 (83.6)	5406 (81.4)	5528 (82.3)	1475 (84.6)	911 (76.8)	3886 (73.8)	848 (75.1)
22	2145 (37.4)	50 (29.9)	125 (32.8)	174 (28.1)	669 (47.4)	352 (36.9)	410 (44.4)	120 (48.8)	27 (17.4)	181 (25.2)	37 (23.7)
23	7724 (80.2)	165 (72.1)	632 (83.6)	747 (78.9)	2094 (84.3)	1252 (82.2)	1296 (82.7)	356 (84.8)	186 (65.5)	828 (71.1)	168 (66.1)
24	11 378 (89.5)	299 (83.8)	962 (92.0)	1135 (89.5)	2845 (90.7)	1843 (90.4)	1806 (90.1)	478 (92.5)	312 (91.0)	1392 (83.7)	306 (89.5)
25	12 419 (91.1)	344 (85.6)	1044 (93.6)	1241 (93.0)	3086 (91.5)	1959 (92.1)	2016 (90.9)	521 (93.0)	386 (95.5)	1485 (86.2)	337 (89.4)
Surfactant overall	11 175 (26.8)	404 (35.0)	1164 (35.3)	1315 (31.5)	1994 (19.2)	2567 (38.6)	1304 (19.4)	810 (46.5)	561 (47.3)	674 (12.8)	382 (33.8)
22	601 (10.5)	22 (13.2)	34 (8.9)	59 (9.5)	119 (8.4)	151 (15.8)	80 (8.7)	74 (30.1)	18 (11.6)	28 (3.9)	16 (10.3)
23	2739 (28.4)	91 (39.7)	307 (40.6)	308 (32.5)	488 (19.7)	640 (42.0)	341 (21.7)	203 (48.3)	113 (39.8)	164 (14.1)	84 (33.1)
24	3891 (30.6)	155 (43.4)	430 (41.1)	456 (36.0)	677 (21.6)	917 (45.0)	428 (21.4)	265 (51.3)	176 (51.3)	248 (14.9)	139 (40.6)
25	3944 (28.9)	136 (33.8)	393 (35.2)	492 (36.9)	710 (21.0)	859 (40.4)	455 (20.5)	268 (47.9)	254 (62.9)	234 (13.6)	143 (37.9)
Assisted ventilation overall	23 225 (55.7)	686 (59.4)	1991 (60.4)	2282 (54.8)	5553 (53.4)	4534 (68.3)	3281 (48.9)	1258 (72.2)	816 (68.8)	2095 (39.8)	729 (64.6)
22	1465 (25.6)	42 (25.1)	84 (22.0)	127 (20.6)	411 (29.1)	301 (31.6)	220 (23.8)	116 (47.2)	27 (17.4)	102 (14.2)	35 (22.4)
23	5524 (57.4)	140 (61.1)	488 (64.6)	527 (55.6)	1368 (55.1)	1066 (70.0)	835 (53.3)	314 (74.8)	176 (62.0)	462 (39.7)	148 (58.3)
24	7864 (61.9)	247 (69.2)	690 (66.0)	797 (62.9)	1800 (57.4)	1550 (76.0)	1086 (54.2)	401 (77.6)	260 (75.8)	770 (46.3)	263 (76.9)
25	8327 (61.4)	257 (63.9)	729 (65.4)	831 (62.2)	1974 (58.5)	1617 (76.0)	1140 (51.4)	427 (76.3)	353 (87.4)	761 (44.2)	283 (75.1)
Ventilation >6 h overall	14 694 (35.2)	447 (38.7)	1474 (44.7)	1639 (39.3)	2797 (26.9)	3060 (46.1)	2044 (30.4)	838 (48.1)	665 (56.1)	1149 (21.8)	581 (51.5)
22	738 (12.9)	24 (14.4)	42 (11.0)	79 (12.8)	177 (12.6)	165 (17.3)	100 (10.8)	67 (27.2)	15 (9.7)	49 (6.8)	20 (12.8)
23	3396 (35.3)	92 (40.2)	350 (46.3)	361 (38.1)	667 (26.9)	704 (46.2)	505 (32.2)	224 (53.3)	140 (49.3)	240 (20.6)	113 (44.5)
24	5051 (39.7)	172 (48.2)	541 (51.7)	568 (44.8)	907 (28.9)	1057 (51.8)	680 (33.9)	264 (51.1)	213 (62.1)	436 (26.2)	213 (62.3)
25	5509 (40.4)	159 (39.6)	541 (48.5)	631 (47.3)	1046 (31.0)	1134 (53.3)	759 (34.2)	283 (50.5)	297 (73.5)	424 (24.6)	235 (62.3)
Antibiotics overall	11 256 (27.0)	332 (28.7)	1382 (41.9)	1365 (32.7)	1828 (17.6)	2490 (37.5)	1343 (20.0)	698 (40.0)	544 (45.9)	889 (16.9)	385 (34.1)
22	593 (10.4)	17 (10.2)	45 (11.8)	64 (10.4)	118 (8.4)	146 (15.3)	75 (8.1)	59 (24.0)	19 (12.3)	35 (4.9)	15 (9.6)
23	2623 (27.2)	67 (29.2)	341 (45.1)	299 (31.6)	448 (18.0)	590 (38.7)	311 (19.8)	172 (41.0)	117 (41.2)	192 (16.5)	86 (33.9)
24	5051 (39.7)	172 (48.2)	541 (51.7)	568 (44.8)	907 (28.9)	1057 (51.8)	680 (33.9)	264 (51.1)	213 (62.1)	436 (26.2)	213 (62.3)
25	4112 (30.2)	128 (31.8)	506 (45.4)	518 (38.8)	637 (18.9)	890 (41.8)	492 (22.2)	253 (45.2)	230 (56.9)	324 (18.8)	134 (35.5)
Cesarean delivery overall	17 860 (42.8)	463 (40.1)	1304 (39.5)	1687 (40.5)	4618 (44.4)	2817 (42.4)	3077 (45.8)	758 (43.5)	451 (38.0)	2219 (42.1)	466 (41.3)
22	511 (8.9)	11 (6.6)	23 (6.0)	26 (4.2)	159 (11.3)	73 (7.7)	104 (11.3)	27 (11.0)	10 (6.5)	65 (9.1)	13 (8.3)
23	3583 (37.2)	72 (31.4)	227 (30.0)	325 (34.3)	980 (39.5)	598 (39.3)	669 (42.7)	172 (41.0)	65 (22.9)	393 (33.7)	82 (32.3)
24	6466 (50.9)	165 (46.2)	491 (46.9)	625 (49.3)	1651 (52.7)	1052 (51.6)	1082 (54.0)	259 (50.1)	162 (47.2)	823 (49.5)	156 (45.6)
25	7300 (53.5)	215 (53.5)	563 (50.5)	711 (53.3)	1828 (54.2)	1094 (51.4)	1222 (55.1)	300 (53.6)	214 (53.0)	938 (54.5)	215 (57.0)
Antenatal steroids overall	17 746 (42.5)	633 (54.8)	1195 (36.2)	1752 (42.0)	4033 (38.8)	3734 (56.2)	2259 (33.6)	1013 (58.1)	676 (57.0)	1949 (37.0)	502 (44.5)
22	982 (17.1)	26 (15.6)	30 (7.9)	98 (15.9)	276 (19.6)	214 (22.5)	121 (13.1)	92 (37.4)	17 (11.0)	82 (11.4)	26 (16.7)
23	4206 (43.7)	126 (55.0)	273 (36.1)	415 (43.8)	1011 (40.7)	906 (59.5)	523 (33.4)	261 (62.1)	161 (56.7)	423 (36.3)	107 (42.1)
24	6009 (47.2)	222 (62.2)	433 (41.4)	587 (46.3)	1306 (41.7)	1248 (61.2)	752 (37.5)	328 (63.4)	218 (63.6)	740 (44.5)	175 (51.2)
25	6549 (48.0)	259 (64.4)	459 (41.2)	652 (48.8)	1440 (42.7)	1366 (64.2)	863 (38.9)	332 (59.3)	280 (69.3)	704 (40.9)	194 (51.5)

Abbreviations: HHS, Department of Health and Human Services; NICU, neonatal intensive care unit.

### Regional Survival

Overall, 26 099 (62.6%) survived infancy with survival rates of approximately 1086 infants at 22 weeks’ (18.9%), 4887 infants at 23 weeks’ (50.8%), 8986 infants at 24 weeks’ (70.7%), and 11140 infants at 25 weeks’(81.7%) of gestational age. There were differences in regional survival rates by gestational age (Table 2). At 22 weeks’ gestation, survival varied by region, from 13 infants in region 8 (8.4%) to 68 infants in region 7 (27.6%). At 23 weeks’ gestation, survival differed by region ranging from 103 infants in region 8 (36.3%) to 890 infants in region 6 (56.7%). Survival at 24 weeks’ gestation ranged between 211 infants in region 8 (61.5%) and 2263 infants in region 4 (72.2%) and 1441 infants in region 6 (71.9%). Survival at 25 weeks’ gestation ranged from 313 infants in region 8 (77.5%) to 344 infants in region 1 (85.6%).

### Active Postnatal Care and Survival

There was moderate positive correlation between regional rates of active care and survival at 22 weeks’ gestation (*r*_τ_[8] = 0.56; *r*^2^ = 0.31; *P* = .03) (Table 3 and Figure). The positive correlation between regional rates of active care and survival at 23 weeks’ gestation was not significant (*r*_τ_[8] = 0.47; *r*^2^ = 0.22; *P* = .07). Rates of active care were not correlated with survival at 24 weeks’ gestation (*r*_τ_[8] = −0.11; *r*^2^ = 0.01; *P* = .73) or 25 weeks’ gestation (*r*_τ_[8] = −0.38; *r*^2^ = 0.14; *P* = .16). Regional rates of NICU admission were positively correlated with regional rates of survival at 22 (*r*_τ_[8] = 0.51; *r*^2^ = 0.26; *P* = .047) but not at 23 to 25 weeks’ gestation. Regional rates of assisted ventilation immediately following delivery were positively correlated at 22 weeks’ gestation (*r*_τ_[8] = 0.51; *r*^2^ = 0.26; *P* = .047) but negatively correlated with survival at 25 weeks’ gestation (*r*_τ_[8] = −0.60; *r*^2^ = 0.36; *P* = .02). In addition, assisted ventilation for more than 6 hours was negatively correlated with survival at 25 weeks’ gestational age (*r*_τ_[8] = −0.64; *r*^2^ = 0.42; *P* = .01). Furthermore, surfactant administration therapy was negatively correlated with survival at 25 weeks’ gestation (*r*_τ_[8] = −0.60; *r*^2^ = 0.36; *P* = .02). There was no correlation between antibiotics for suspected sepsis and survival at any gestational age by region. All data for the presence of seizures were suppressed and therefore not estimated.

**Table 3.** Correlations Between Regional Gestational Age-Specific Survival and Regional Rates of Active Postnatal Care and Perinatal Care

Type of care, by gestational age in wk	τ	*R* ^2^	*P* value
Active care			
22	0.56	0.31	.03^a^
23	0.47	0.22	.07
24	–0.11	0.01	.78
25	–0.38	0.14	.16
NICU Admission			
22	0.51	0.26	.046^a^
23	0.47	0.22	.07
24	–0.11	0.01	.73
25	–0.47	0.22	.07
Surfactant			
22	0.07	<0.01	.86
23	–0.07	<0.01	.86
24	–0.64	0.42	.01^a^
25	–0.60	0.36	.02^a^
Assisted ventilation			
22	0.51	0.26	.046^a^
23	–0.11	0.01	.73
24	–0.38	0.14	.16
25	–0.60	0.36	.02^a^
Ventilation >6 h			
22	0.29	0.08	.29
23	–0.16	0.02	.60
24	–0.33	0.11	.22
25	–0.64	0.42	.01^a^
Antibiotics			
22	–0.16	0.02	.60
23	–0.16	0.02	.60
24	–0.38	0.14	.16
25	–0.42	0.18	.11
Antenatal steroids			
22	0.60	0.36	.02^a^
23	–0.20	0.04	.48
24	–0.69	0.47	.004^a^
25	–0.24	0.06	.38
Cesarean delivery			
22	0.47	0.22	.07
23	0.73	0.54	.002^a^
24	0.07	<0.01	.86
25	0.02	<0.01	>.99

Abbreviation: NICU, neonatal intensive care unit.

^a^ Significance was set at *P* < .05.

**Figure.**
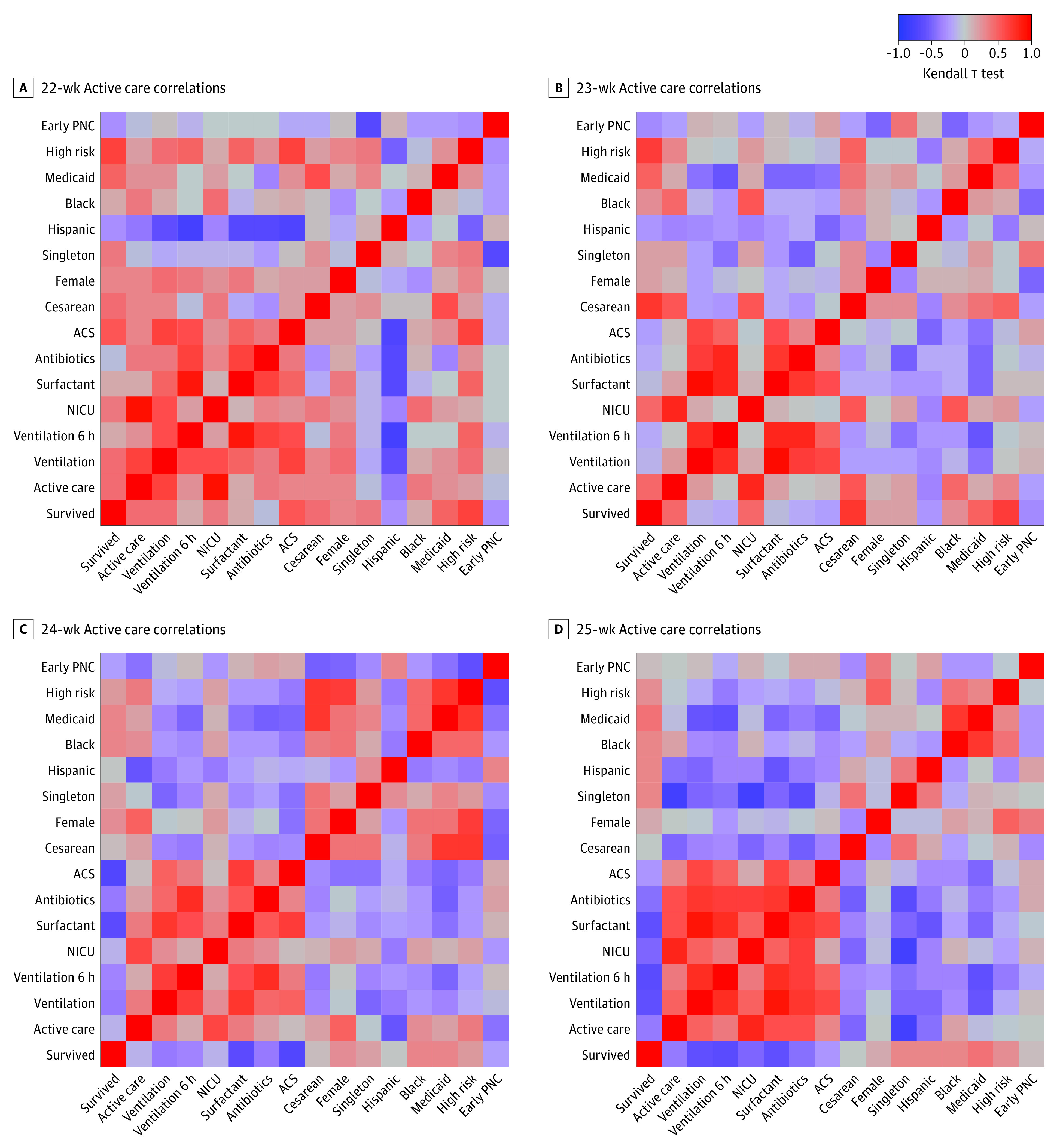
Correlation Between Frequency of Active Care and Survival Rate Among Periviable Infants Collapsed regional-level data was obtained from the US Centers for Disease Control and Prevention WONDER linked live birth and infant death expanded database from 2017 to 2020. This was used to compare the correlation between the regional rate of survival, care practices, and baseline characteristics among infants at 22, 23, 24, and 25 weeks’ gestation. Active care was defined as the use of 1 or more of the following: neonatal intensive care unit (NICU) admission, surfactant replacement therapy, assisted ventilation, antibiotics for suspected neonatal sepsis, and seizures. Heat maps were created using Kendall τ correlation regression analysis with red indicating positive correlations, gray representing no/weak correlations, and blue representing negative correlations. ACS indicates antenatal corticosteroids; PNC, prenatal care.

### Active Perinatal Care and Survival

Rates of cesarean delivery varied by gestational age from 511 infants (8.9%) (regional range, 4.2% to 11.3%) at 22 weeks’ gestation to 7300 infants (53.5%) (regional range, 50.5% to 57.0%) at 25 weeks’ gestation (Table 2). Regional rates of cesarean delivery rates were associated with survival at 23 weeks’ gestation (*r*_τ_[8] = 0.73; *r*^2^ = 0.54; *P* = .002) (Table 3 and Figure). Rates of cesarean were not correlated with regional survival differences at 22, 24, or 25 weeks’ gestation. Reported rates of antenatal corticosteroid administration were 982 infants (17.1%; range, 7.9% to 37.4%) at 22 weeks’ gestation and 6549 infants (48.0%; range, 38.9% to 69.3%) at 25 weeks’ gestation. There was a moderate positive correlation at 22 weeks’ gestation (*r*_τ_[8] = 0.60; *r*^2^ = 0.36; *P* = .02) and a moderate negative correlation at 24 weeks’ gestation (*r*_τ_[8] = −0.69; *r*^2^ = 0.47; *P* = .004) between reported regional rates of antenatal corticosteroid administration and survival.

### Additional Analyses

Regional rates of survival were correlated with regional rates of Medicaid insurance at 22, 23, and 25 weeks’ gestation (Table 3 and Figure). Regional rates of higher-risk pregnancy were also correlated with survival at 22 and 23 weeks’ gestation. Regional rates of early prenatal care, sex, singleton births, non-Hispanic Black births, and Hispanic births were not correlated with gestational-age specific survival rates (Table 4). Regional survival at 22 weeks’ gestation was strongly positively correlated with survival at 23 weeks’ gestation (*r*_τ_[8] = 0.82; *r*^2^ = 0.68; *P* < .001) (eTable in Supplement 1). Regional survival rates at 22 or 23 weeks’ gestation were not correlated with survival rates at 24 or 25 weeks’ gestation.

**Table 4.** Correlations Between Regional Gestational Age-Specific Survival and Regional Rates of Baseline Characteristics

Characteristic, by gestational age in wk	τ	*R* ^2^	*P* value
Female			
22	0.47	0.22	.07
23	0.20	0.04	.48
24	0.29	0.08	.29
25	0.16	0.02	.60
Singleton			
22	0.11	0.01	.73
23	0.20	0.04	.48
24	0.20	0.04	.48
25	0.33	0.11	.22
Medicaid insurance			
22	0.73	0.54	.002^a^
23	0.56	0.31	.03^a^
24	0.38	0.14	.16
25	0.56	0.31	.03^a^
Higher-risk pregnancy			
22	0.67	0.44	.01^a^
23	0.69	0.47	.004^a^
24	0.24	0.06	.38
25	0.29	0.08	.29
Early prenatal care			
22	–0.24	0.06	.38
23	–0.29	0.08	.29
24	–0.20	<0.01	.48
25	0.07	<0.01	.86
Hispanic			
22	–0.20	0.04	.48
23	–0.24	0.06	.38
24	0.02	<0.01	>.99
25	0.33	0.11	.22
Non-Hispanic Black			
22	0.33	0.11	.22
23	0.29	0.08	.29
24	0.33	0.11	.22
25	0.33	0.11	.22

^a^ Significance was set at *P* < .05.

## Discussion

The results of this study supported our hypothesis that regions with higher rates of active postnatal care have higher gestational age-specific survival rates among periviable infants. Active postnatal care and neonatal intensive care unit admission among infants at the lowest gestations differ by region in the US. In addition, there are marked differences in survival among periviable infants by region in the US. Much of the variation in survival at 22 weeks’ gestation within the US is explained by regional differences in the provision of active postnatal care.

Additional antenatal factors other than gestational age are associated with prognosis among periviable infants. Not only are larger infants more likely to receive intensive care, but higher birth weight has been associated with higher rates of survival without profound neurodevelopmental impairment.^1^ Professional guidelines are increasingly moving away from decision-making based solely on gestational age and instead are advocating decision-making according to stratified local and individual risks. The British Association of Perinatal Medicine guidelines recommend comfort-focused care for infants with greater than 90% risk of death or severe impairment, individualized care according to parental preferences with 50% to 90% risk of death or severe impairment, and active care for infants with less than 50% risk of death or severe impairment.^17^ Therefore, differences in rates of survival among periviable infants at a local and regional level may affect perinatal decision-making. However, it is also possible that perinatal decision-making at a local or regional level becomes a so-called self-fulfilling prophecy leading to differences in survival among periviable infants.^18^

The results of our study agree with the results of a study reporting that differences in survival across 24 US academic centers were in part explained by differences in the initiation of active postnatal care among infants born at 22, 23, and 24 weeks’ gestation.^8^ Overall, among infants receiving active postnatal care, 65.0% survived, and 56.1% survived without severe neurodevelopmental impairment. In contrast, all infants who did not receive active treatment died on the day of birth.^8^ Our study expanded the generalizability of these findings by using a national database which allowed us to analyze by US region. However, we did not have access to data regarding longer-term outcomes, including neurodevelopmental outcomes, which are a major concern to families and physicians when considering whether to initiate or forgo active care in this patient population.

Although the risk of adverse neurodevelopmental outcomes, including cognitive impairment, motor impairment, cerebral palsy, bilateral deafness, and blindness may be a concern, recent studies indicate that improved survival in periviable infants does not correlate with increasing rates of neurodevelopmental impairment.^19,20^ For example, among Swedish extremely preterm infants receiving active prenatal care, 73% had no or mild neurodevelopmental impairment at 2.5-year follow-up and outcomes improved with each week of gestational age.^19^ A 2017 study that used data from 11 academic health care centers in the US reported that the improvement in the rate of survival among periviable infants was not associated with a disproportionate increase in survival with neurodevelopmental impairment.^20^

Our study also agrees with the aforementioned studies from Sweden and the US on regional differences in periviable care. It is likely that differences in the receipt of perinatal care practices,^11,12,13^ including rates of cesarean as noted in our study, may be a marker for the aggressiveness of perinatal interventions on behalf of the infant.^21^ This, in turn, may translate into early differences in survival associated with the provision of intensive care.^8,11,22,23^ We found a negative correlation between assisted ventilation and surfactant with survival at 24- and 25-weeks’ gestation. This is consistent with data from randomized clinical trials suggesting that early CPAP and selective surfactant therapy is superior to prophylactic surfactant and mechanical ventilation among infants at 24 or more weeks’ gestation.^24^ Reported rates of antenatal corticosteroid administration were low in this study compared with the Vermont Oxford Network,^25^ and these data likely reflect the inaccuracy of antenatal corticosteroid reporting on birth certificates.^26^ Our study did not assess the quality of birth certificate data in the US,^27,28^ but the high rate of active postnatal care at 24 and 25 weeks’ gestation, which would be expected at these later gestational ages, supports the validity of our definition.

We noted an association between gestational-age specific survival at 22 weeks’ gestation and survival at 23 weeks’ gestation. It has been previously noted that the center-level approach to care among infants at the lowest gestations has a positive effect for survival and major morbidities among more mature infants.^29^ We did not directly examine racial/ethnic differences in the provision of active care at the lowest gestations. Lower rates of active postnatal care among non-Hispanic Black infants compared with White infants have been reported.^8^ It is also known that state-level differences in infant mortality rate vary by the proportion of Black births in each state.^10^ Our study did not examine how regional differences in racial and ethnic composition, socioeconomic status, or cultural perspectives are associated with postnatal care. Furthermore, the definition of race and ethnicity we reported was simplistic compared with the potential 31 categories available in the database.

### Limitations

This study has limitations. We assessed rates of active care and their correlation with periviable infant survival in the 10 HHS regions, but there may be important differences between states as well as individual hospitals within these regions that were not controlled for in the current study. However, as many states have small numbers of infants at the lowest gestations, these data would be suppressed per National Center for Health Statistics rules. Furthermore, we did not have access to data on individual mother-infant dyads, which could have provided greater insight into the role active care plays in survival. We did not include fetal deaths in this study and the impact of differences in the reporting of fetal deaths was not examined.^30,31^ As fetal death rates are higher among states in the southeast region of the US, which had higher rates of active care in our study, it is unlikely that differences in reporting of fetal deaths contributed significantly. In addition, we excluded previable births that account for much of the variability in fetal death reporting from this study. Furthermore, we excluded infants less than 400 g who are at highest risk of adverse outcomes.^32^ It is possible that some infants who died without meeting our definition of active care received some limited resuscitation attempts before the decision was made to withdraw care.^8^ In this population-based study, we did not adjust for regional differences in baseline characteristics, so it is possible there is some bias we did not account for in our estimates.

## Conclusions

In this cohort study of 10 US regions including 41 707 periviable infants, regional differences in measures of active postnatal care, including neonatal intensive care unit admission and assisted ventilation, were associated with higher survival rates at 22 weeks’ gestation in US regions. Results should be interpreted with caution as individual patient-level data were not available.

## Notes

Supplement 1. **eTable.** Correlation Between Regional Gestational Age-Specific Survival at Lower vs Higher Gestational Ages

Supplement 2.
Data Sharing Statement
