# Supplementary material for: Association of Active Postnatal Care With Infant Survival Among Periviable Infants in the US
Source: JAMA Netw Open. 2023 Jan 19;6(1):e2250593. doi: 10.1001/jamanetworkopen.2022.50593 (PMC9856598; doi:10.1001/jamanetworkopen.2022.50593)
Supplement: Supplement 1. — eTable. Correlation Between Regional Gestational Age-Specific Survival at Lower vs Higher Gestational Ages [file jamanetwopen-e2250593-s001.pdf]

Supplemental Online Content

Silva ER, Shukla VV, Tindal R, Carlo WA, Travers CP. Association of active postnatal care with infant survival among periviable infants in the US. *JAMA Netw Open*. 2023;6(1):e2250593. doi:10.1001/jamanetworkopen.2022.50593

eTable. Correlation Between Regional Gestational Age-Specific Survival at Lower vs Higher Gestational Ages

This supplemental material has been provided by the authors to give readers additional information about their work.

© 2023 Silva ER et al. *JAMA Network Open.*

eTable. Correlation Between Regional Gestational Age-Specific Survival at Lower vs Higher Gestational Ages

	Tau	R^2	p value
22 versus 23	0.82	0.68	<0.001*
22 versus 24	0.29	0.08	0.29
22 versus 25	0.02	<0.01	1.00
23 versus 24	0.47	0.22	0.07
23 versus 25	-0.07	<0.01	0.86
24 versus 25	0.20	0.04	0.48

*Significant at P <0.05.

© 2023 Silva ER et al. *JAMA Network Open.*
