# Supplementary material for: Association of Active Postnatal Care With Infant Survival Among Periviable Infants in the US
Source: JAMA Netw Open. 2023 Jan 19;6(1):e2250593. doi: 10.1001/jamanetworkopen.2022.50593 (PMC9856598; doi:10.1001/jamanetworkopen.2022.50593)
Supplement: Supplement 2. — Data Sharing Statement [file jamanetwopen-e2250593-s002.pdf]

Data Sharing Statement

Silva. Association of Active Postnatal Care With Infant Survival Among Periviable Infants in the US. *JAMA Netw Open*. Published January 19, 2023.
doi:10.1001/jamanetworkopen.2022.50593

Data
Data available: No

Additional Information
Explanation for why data not available: The data used in this study are publically available on CDC WONDER and can be accessed using the following link:
https://wonder.cdc.gov/controller/datarequest/D159
